# The Relationship Between U.S. East Coast Sea Level and the Atlantic Meridional Overturning Circulation: A Review

**DOI:** 10.1029/2019JC015152

**Published:** 2019-09-04

**Authors:** Christopher M. Little, Aixue Hu, Chris W. Hughes, Gerard D. McCarthy, Christopher G. Piecuch, Rui M. Ponte, Matthew D. Thomas

**Affiliations:** ^1^ Atmospheric and Environmental Research, Inc. Lexington MA USA; ^2^ Climate and Global Dynamics Laboratory National Center for Atmospheric Research Boulder CO USA; ^3^ Department of Earth, Ocean and Ecological Sciences University of Liverpool Liverpool UK; ^4^ National Oceanography Centre Liverpool UK; ^5^ ICARUS, Department of Geography Maynooth University Maynooth Ireland; ^6^ Woods Hole Oceanographic Institution Woods Hole MA USA; ^7^ Department of Geology and Geophysics Yale University New Haven CT USA

**Keywords:** sea level, AMOC, United States, coastal, climate model, review

## Abstract

Scientific and societal interest in the relationship between the Atlantic Meridional Overturning Circulation (AMOC) and U.S. East Coast sea level has intensified over the past decade, largely due to (1) projected, and potentially ongoing, enhancement of sea level rise associated with AMOC weakening and (2) the potential for observations of U.S. East Coast sea level to inform reconstructions of North Atlantic circulation and climate. These implications have inspired a wealth of model‐ and observation‐based analyses. Here, we review this research, finding consistent support in numerical models for an antiphase relationship between AMOC strength and dynamic sea level. However, simulations exhibit substantial along‐coast and intermodel differences in the amplitude of AMOC‐associated dynamic sea level variability. Observational analyses focusing on shorter (generally less than decadal) timescales show robust relationships between some components of the North Atlantic large‐scale circulation and coastal sea level variability, but the causal relationships between different observational metrics, AMOC, and sea level are often unclear. We highlight the importance of existing and future research seeking to understand relationships between AMOC and its component currents, the role of ageostrophic processes near the coast, and the interplay of local and remote forcing. Such research will help reconcile the results of different numerical simulations with each other and with observations, inform the physical origins of covariability, and reveal the sensitivity of scaling relationships to forcing, timescale, and model representation. This information will, in turn, provide a more complete characterization of uncertainty in relevant relationships, leading to more robust reconstructions and projections.

## Key Points

- The relationship between the AMOC and coastal sea level is important to flood risk projections and ocean circulation reconstructions
- The amplitude and pattern of sea level variability associated with AMOC variations is location, forcing, timescale, and model dependent
- Future research should address the complex spatiotemporal structure of AMOC and the role of near‐coast ageostrophic processes

## 1 Sea Level Variability Along the United States East Coast and Its Societal Importance

**Figure 1:**
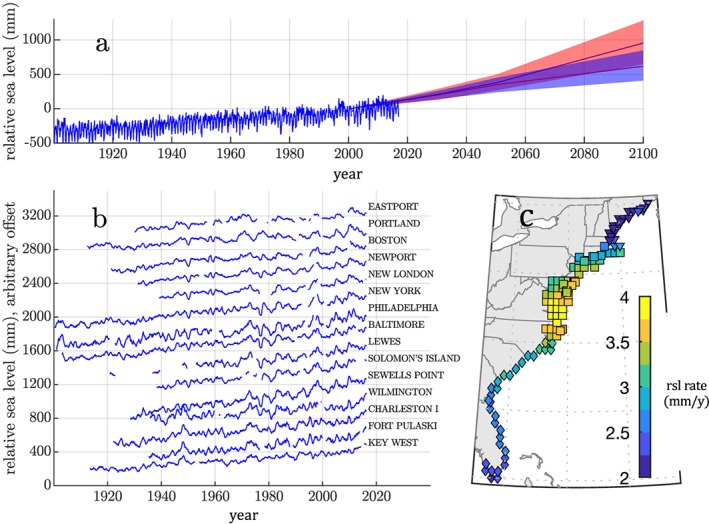
(a) Monthly mean tide gauge sea level (in millimeters relative to year 2000) at the Battery (New York City; blue line). Projections of relative sea level (RSL) change, relative to year 2000, for RCP 2.6 (blue) and RCP 8.5 emission scenarios (red; Kopp et al., 2014). Shading after the year 2000 indicates 17th to 83rd percentile range of RSL projections. (b) Annual mean RSL (in millimeters, with arbitrary offset) measured at 15 U.S. East Coast tide gauges (Holgate et al., 2013) with long and relatively complete records. (c) Linear trend in RSL along the U.S. East Coast from 1900–2017, in millimeters per year, from a Bayesian reconstruction (panel taken from Piecuch, Huybers, et al., 2018).

The densely populated U.S. East Coast is especially vulnerable to the impacts of sea level change, with ~2.4 million people and ~1.4 million housing units between Maine and Florida less than 1 m above local mean high water (Strauss et al., 2012). Here, sea level rise is already having adverse environmental, societal, and economic consequences, including increases in the severity and frequency of coastal flooding (e.g., Ezer & Atkinson, 2014; Moftakhari et al., 2015; Ray & Foster, 2016; Sweet et al., 2018; Wdowinski et al., 2016). Regional rates of sea level rise, and their associated consequences, are projected to increase substantially over the coming century (Figure 1a; Brown et al., 2018; Dahl et al., 2017; Kopp et al., 2014; Little, Horton, Kopp, Oppenheimer, Yip, 2015; Ray & Foster, 2016; Vitousek et al., 2017).

Understanding the drivers of future change in relative sea level (RSL, i.e., that observed by tide gauges and relevant to coastal locations; see Gregory et al., 2019), and the ability of numerical models to represent such drivers, is critical. However, this is a complex task, given the many contributing processes that operate over different temporal and spatial scales, including, for example: freshwater input from land and the cryosphere, thermal expansion of sea water, glacial isostatic adjustment, and oceanic mass and volume redistribution (see Kopp et al., 2015; Milne et al., 2009; Stammer et al., 2013, for more thorough reviews of these processes).

The relative contributions of these processes to U.S. East Coast RSL vary across space and through time. For example, vertical land motion (due primarily to glacial isostatic adjustment) accounts for the majority of the large‐scale spatial variation in recent centennial trends and underlies the high rates of RSL rise in the Mid‐Atlantic (Figure 1c; Karegar et al., 2017; Piecuch, Huybers, et al., 2018). However, ongoing climate‐related processes—associated with net freshwater input, atmosphere‐ocean momentum and buoyancy fluxes, and ocean mass and volume redistribution—dominate the interannual to multidecadal, spatially variable, U.S. east coast RSL signals during the twentieth century (Figure 1b; Andres et al., 2013; Bingham & Hughes, 2009; Davis & Vinogradova, 2017; Ezer, 2013; Ezer et al., 2013; Frederikse et al., 2017; Goddard et al., 2015; Park & Sweet, 2015; Piecuch et al., 2016; Piecuch, Bittermann, et al., 2018; Piecuch & Ponte, 2015; Thompson & Mitchum, 2014; Woodworth et al., 2014; Yin & Goddard, 2013).

Of interest in this review paper is RSL variability related to changes in ocean circulation and density that may be causally coupled, or simply correlated, with the Atlantic Meridional Overturning Circulation (AMOC; see section 2). We thus focus on variability in “dynamic sea level” (DSL), that is, the height of the sea surface above the geoid, with the inverse barometer correction applied (Gregory et al., 2019). Secular DSL changes are evident in 21st century climate model simulations and are projected to be a principal driver of acceleration in 21st century sea level and its spatial variation along the east coast (Bilbao et al., 2015; Bouttes et al., 2014; Carson et al., 2016; Chen et al., 2018; Church et al., 2013; Kopp et al., 2014; Little, Horton, Kopp, Oppenheimer, Vecchi, et al., 2015; Little, Horton, Kopp, Oppenheimer, & Yip, 2015; Perrette et al., 2013; Slangen et al., 2014; Yin et al., 2009; Yin, 2012; Yin & Goddard, 2013). Various studies have shown these large‐scale regional DSL anomalies to be correlated with a decline in AMOC strength (section 4). However, current‐generation climate models also show a wide range in future projections of regional DSL rise. They may also exhibit systematic biases due to poorly resolved processes that influence near‐coast DSL (section 6).

An improved theoretical and observational basis for AMOC‐DSL relationships would enable assessments of the reliability of individual model projections, and climate models more generally, allowing improved estimates of the magnitude, spatial pattern, and time of emergence of expected sea level rise. In addition, a robust “signature” of AMOC (or some other feature of the large‐scale circulation) in coastal RSL could be leveraged to infer preinstrumental changes in AMOC and/or climate. Recent improvements to analysis of the tide gauge record, including approaches to cope with data gaps and account for vertical land motion and glacial isostatic adjustment (Kopp, 2013; Piecuch, Huybers, et al., 2017), have intensified the interest in exploiting this relationship to inform reconstructions of ocean variability (e.g., Butler et al., 2015; Kienert & Rahmstorf, 2012; McCarthy et al., 2015). Proxies that predate the tide gauge record offer the opportunity to extend these reconstructions over centennial to millennial timescales (e.g., Kemp et al., 2017, 2018).

Here, motivated by these considerations, we review evidence for the covariation of AMOC and U.S. East Coast sea level. In section 2, we define AMOC and its relationship to the large‐scale circulation of the North Atlantic Ocean. Section 3 presents a simple diagnostic scaling argument between AMOC strength and DSL. Section 4 surveys AMOC‐DSL linkages in numerical simulations (where long‐period relationships are able to be assessed) and includes a new analysis of the AMOC‐DSL scaling coefficient in Coupled Model Intercomparison Project Phase 5 (CMIP5) simulations. Section 5 examines observational linkages between AMOC components and coastal sea level, clarifying the specific components of AMOC (e.g., Gulf Stream) invoked, the regional fingerprint of such linkages, and the timescales over which the relationship has been documented. In section 6, we suggest potential origins of along‐coast variations, intersimulation differences in scaling relationships, and discrepancies between models and observations; section 7 highlights new research directions that can help assess these discrepancies more extensively and quantitatively.

## 2 AMOC and the North Atlantic Ocean Circulation

**Figure 2:**
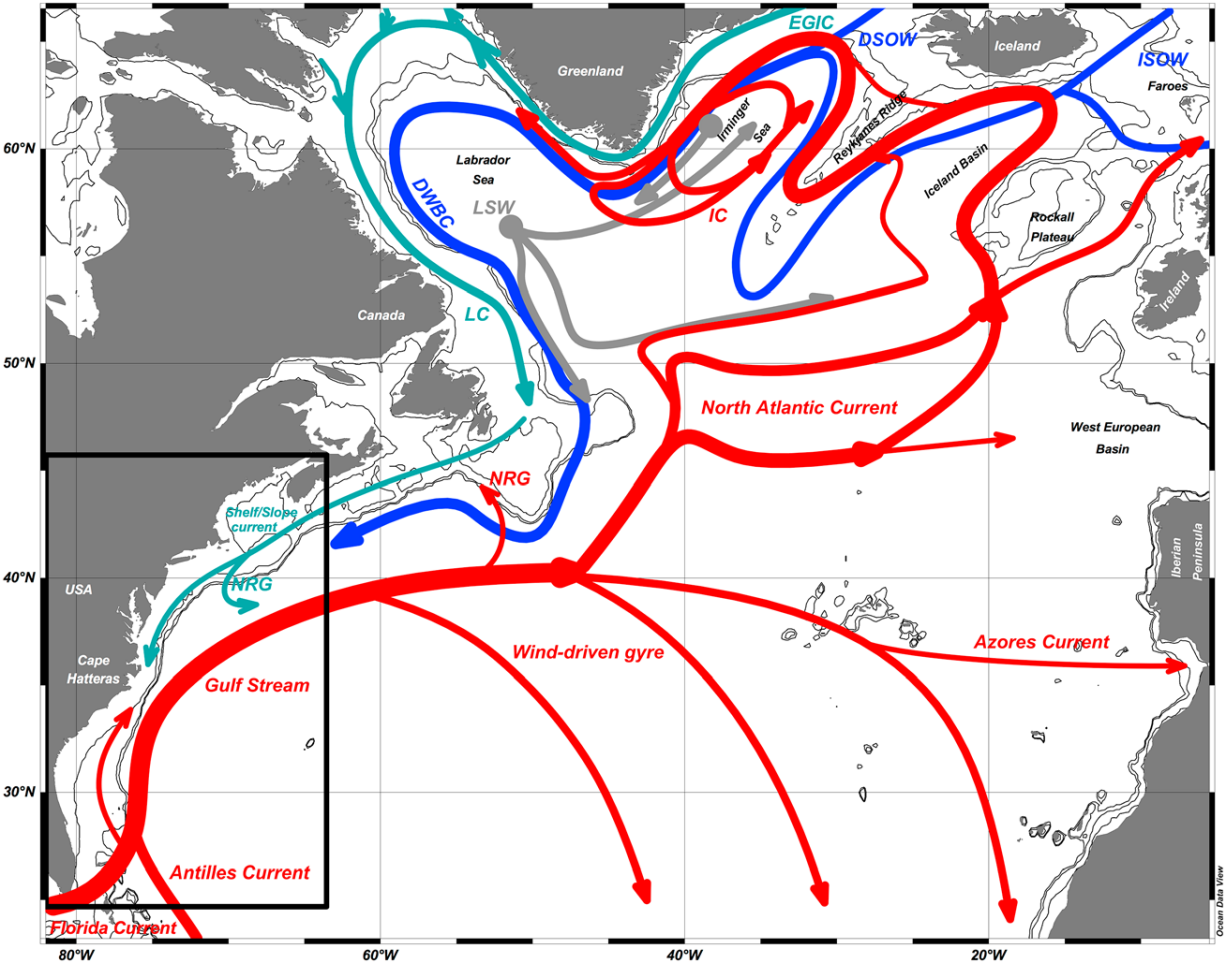
Schematic of key AMOC‐related components of the North Atlantic Ocean (modified from García‐Ibáñez et al., 2018). Abbreviations are as follows: NRG = Northern Recirculation Gyre; LC = Labrador Current; DWBC = Deep Western Boundary Current; IC = Irminger Current; EGIC = East Greenland‐Irminger Current. Three source waters for North Atlantic Deep Water are noted: LSW = Labrador Sea Water; ISOW = Iceland‐Scotland Overflow Water; DSOW = Denmark Straits Overflow Water. Box indicates the U.S. East Coast region.

The U.S. East Coast borders the western boundary of the North Atlantic Ocean, which is characterized by a spatially and temporally complex system of surface and deep currents (Figure 2).

At U.S. East Coast latitudes, the large‐scale ocean circulation is dominated by two opposing gyres. At subtropical latitudes, southward wind‐driven transport in the interior of the gyre is closed by a western boundary current, composed of the Gulf Stream to the north and the Florida and Antilles currents further south. At subpolar latitudes, the North Atlantic Current (NAC) splits into various branches that flow northwards along the eastern side of the subpolar gyre (Rhein et al., 2011). These currents flow cyclonically around the subpolar gyre, contributing to the upper parts of the western boundary currents comprising the East and West Greenland Currents and the Labrador Current. Part of the NAC also flows into the Nordic Seas (e.g., Dickson & Brown, 1994; Sarafanov et al., 2012). Along these high‐latitude branches, warm and salty surface waters originating from the tropical and subtropical Atlantic increase in density and transform into North Atlantic Deep Water through a variety of processes, including cooling, mixing, and convection (Marotzke & Scott, 1999; Spall & Pickart, 2001; Thomas et al., 2015).

In addition to these large‐scale flows, there are important currents along the U.S. East Coast continental shelf, shelf break, and slope: flowing northward over the continental shelf south of Cape Hatteras (the South Atlantic Bight) and southward along the shelf between Cape Hatteras and Nova Scotia (Figure 2). These currents are driven by a combination of local wind and buoyancy forcing as well as interactions with the larger‐scale flow field (see section 6). In the South Atlantic Bight, interactions between the shelf current and the Gulf Stream are clearly important, but there is evidence of locally wind driven variability closer to the shore (Lee et al., 1991; Stegmann & Yoder, 1996; Yuan et al., 2017). To the north of Cape Hatteras, the Slope Current has its origins in the Labrador Current and the East Greenland Current (Chapman & Beardsley, 1989; Rossby et al., 2014). Its strength is therefore linked to the AMOC, through the strength of the Labrador Current, as well as through interactions with the Northern Recirculation Gyre (Andres et al., 2013; Zhang, 2008), the Deep Western Boundary Current (e.g. Zhang & Vallis, 2007), and the Gulf Stream (Ezer, 2015).

**Figure 3:**
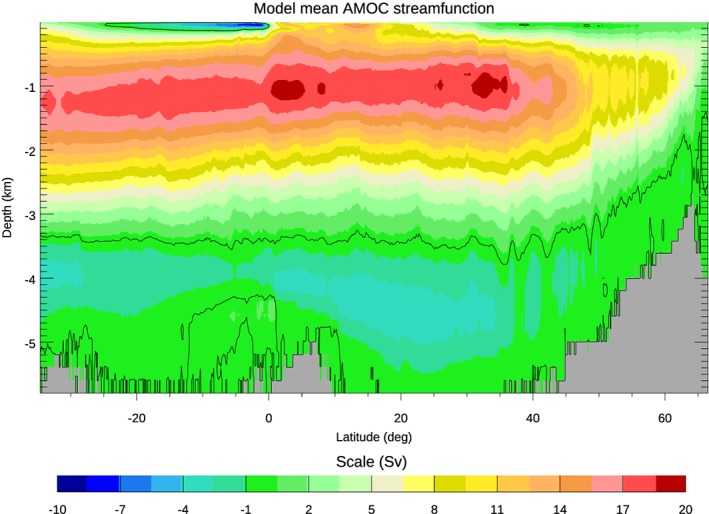
The AMOC, averaged over the 1959–2012 period, from a 1/12° resolution model simulation as described in Hughes et al. (2018). The flow is clockwise around positive values, and the stream function is calculated by integrating the southward velocity both zonally and upwards from the bottom. The black contour is at zero.

In aggregate, these horizontal and vertical flows result in an “overturning” circulation that transports over 1 PW of heat poleward (Trenberth & Fasullo, 2017). In this paper, this AMOC is defined as the stream function of the zonally and cumulatively vertically integrated meridional velocity of the Atlantic Ocean north of 35°S (Buckley & Marshall, 2016; Zhang, 2010). In models and observations, the AMOC reveals upper and lower interhemispheric overturning cells of water that are sourced by high‐latitude sites of deep water formation in the northern and southern hemispheres respectively (Figure 3).

The upper overturning cell reflects northward transport in the upper ocean currents, including those mentioned earlier in this section, compensated by southward flowing North Atlantic Deep Water at intermediate depths. In models, the maximum of the AMOC stream function is typically located around the latitude of the Gulf Stream separation and at approximately 1,000‐m depth. Below this upper cell is a lower cell of Antarctic Bottom Water that originates from sources at high southern latitudes (Buckley & Marshall, 2016; Kuhlbrodt et al., 2007). See Buckley and Marshall (2016) and other reviews in this special issue, particularly Bower et al. (2019), for a more comprehensive description of AMOC structure and variability.

## 3 A Simple Theoretical Basis for AMOC‐DSL Covariability

A diagnostic relationship between the AMOC and DSL can be derived from the zonal momentum equation:

(1) $$\frac{\rho }{r\cos\phi }\frac{D}{\mathrm{Dt}}\left(\mathrm{ur}\cos\phi \right)-\mathrm{\rho fv}+\mathrm{\rho fw}\cot\phi =-\frac{1}{r\cos\phi }\frac{\partial p}{\partial \lambda }+F_{x},$$

where *r* is the Earth's radius, *u* is the zonal velocity, *v* is the meridional velocity, *w* is the vertical velocity, *f* is the Coriolis frequency, ϕ is latitude, λ is longitude, *p* is pressure, *ρ* is density, *F*_*x*_ is the eastward viscous force per unit volume, and *D/Dt* is the material rate of change. For a derivation and discussion of the equations of motion see, for example, Vallis (2006, Chapter 2) and Gill (1982, Chapter 4). If we (1) zonally integrate over the basin and (2) neglect the advection of relative angular momentum (the first term), the term involving *w* (usually neglected in the Primitive Equations), and the viscous term (assuming we are below the surface Ekman layer, and that any bottom Ekman layer occupies only a small fraction of the zonal integral—this assumes that we are at depths where it is meaningful to consider the ocean to have sidewalls), this reduces to an integrated geostrophic balance:

(2) $$\text{fT}=p_{E}-p_{W},$$

where T is the northward mass transport across the section (the zonal integral of *ρv*). Equation (2) relates the northward mass transport to the difference between pressure at the eastern end (*p*_*E*_) and the western end (*p*_*W*_) of the section. These pressures are bottom pressures, which become equivalent to DSL (with a scaling of approximately 1 cm/mbar of pressure) as the depth tends to zero at the coast.

This zonally integrated geostrophic balance can be used to derive a simple scaling between the AMOC and DSL at the western boundary. First, we note that the eastern boundary pressure is very close to being a function of depth alone, independent of latitude, at least below a depth of around 100 m (Hughes et al., 2018; Hughes & de Cuevas, 2001). Subtracting off this reference function of depth in our definition of *p* (which now should be considered to be a pressure anomaly, referenced to the eastern boundary value), we find that *p*_*E*_ = 0. Then, integrating over depth from the surface (*z* = 0) to the depth of the maximum in the overturning streamfunction (*z* =  − *H*), we find that the total northward mass transport above this depth is given by

(3) $$Q=\int_{-H}^{0}T\;dz=-\frac{1}{f}\int_{-H}^{0}p_{W}\;dz=-\frac{H}{f}\bar{p_{W}},$$

where $\bar{p_{W}}$ is the western boundary pressure averaged over the depth range above the maximum overturning. The relationship to coastal sea level then follows from the assumption that the depth‐averaged pressure in this zone is related to the boundary pressure near the surface, *p*_*W*0_, which is in turn related to inverse barometer‐corrected boundary sea level *h*_*W*_ by *ρ*_0_*gh*_*W*_ = *p*_*W*0_, where we use a reference density *ρ*_0_. Rewriting in terms of this near‐surface western boundary pressure anomaly, we find

(4) $$Q=-\frac{H_{e}}{f}p_{W0}=-\frac{H_{e}}{f}\rho_{0}gh_{W},$$

requiring the definition of an effective layer thickness

(5) $$H_{e}=\int_{-H}^{0}\frac{p_{W}}{p_{W0}}\;dz,$$

which may be interpreted as the layer thickness used to multiply the near‐surface boundary pressure anomaly (proportional to sea level), in order to get the correct depth‐integrated pressure force on the sidewall. If the pressure anomaly (or equivalently the northward transport) is independent of depth above −*H*, *H*_*e*_ = *H*. If the zonally integrated flow (or pressure anomaly) is largest at the surface and decreases linearly to zero at the maximum of the overturning, *H*_*e*_ = 0.5*H*. Rearranging (5), we find that the coastal sea level signal can be written as

(6) $$h_{W}=-\frac{Q}{\rho_{0}}\frac{f}{{\mathrm{gH}}_{e}},$$

in which it is shown how the coastal sea level signal *h*_*W*_ is negatively related to the strength of the overturning *Q*/*ρ*_0_, and the size of the signal is larger if the effective layer thickness *H*_*e*_ is smaller.

Figure 3 reveals a fairly uniform (or slowly decreasing with increasing depth) northward zonally integrated flow above about 1,000‐m depth, balanced by a deeper return flow (with more complicated flows in the top few hundred meters, representing the wind‐driven flow superimposed on the large‐scale MOC). Assuming *f =* 10^−4^ s^−1^ (true at a latitude of about 43°N), equation (6) predicts a sea level change of 1 cm/Sv of meridional transport (less for latitudes closer to the equator, and slightly more for more poleward latitudes). If, rather than constant transport per unit depth above 1,000 m (as in a simple two‐layer model), we assume a linear rise from zero at 1,000 m to a maximum at the surface, then pressure at the surface is twice the depth average, leading to a scaling of −2 cm/Sv. Realistic scalings are likely to be between these limits, subject to the assumption of geostrophic balance in equation (2), and the approximation that the vertical profile of the flow remains constant (temporal variations in *H*_*e*_ are proportionally smaller than those in *Q*). The dependence on *f* means that this scaling should also lead to smaller sea level signals closer to the equator, again assuming that proportional variations in *H*_*e*_ are smaller than those in *f*.

## 4 Evidence of an AMOC‐DSL Relationship in Numerical Models

Numerical simulations allow analysis of AMOC‐DSL relationships that can be compared to the theoretical considerations of the previous section, while incorporating local and large‐scale forcing, complex 3‐D flows, and ageostrophic processes, to the extent permitted by their resolution. Most analysis of numerical simulations has focused on 21st century, centennial‐timescale, AMOC‐DSL relationships. In this section, we thus focus on longer timescales, although we contrast these results with selected studies that have examined covariability over shorter timescales, often with a focus on the historical record.

The connection between U.S. East Coast sea level rise and the AMOC in coupled climate models was first established by Levermann et al. (2005) through “hosing” simulations (in which extreme freshwater forcing is applied to the subpolar North Atlantic). They found that, in a climate model with a relatively coarse (3.75° horizontal resolution) ocean, a weakened AMOC is associated with DSL rise in most of the Atlantic basin, with a scaling coefficient of up to −5 cm/Sv. Most subsequent numerical simulations that have assessed this relationship show a more complex spatial pattern of DSL change (e.g., Kienert & Rahmstorf, 2012; Landerer et al., 2007; Lorbacher et al., 2010; Yin et al., 2010), and a smaller (less negative) scaling coefficient (e.g., Bingham & Hughes, 2009; Little et al., 2017; Schleussner et al., 2011). However, the correlation between DSL rise over portions of the U.S. East Coast and a decline in AMOC (and, often, a rise in steric height in the western North Atlantic intergyre region) has been repeatedly noted, in simulations forced by future greenhouse gas emission scenarios, freshwater input into the subpolar North Atlantic, or both (e.g., Hu et al., 2009; Hu et al., 2011; Hu & Bates, 2018; Hu & Deser, 2013; Kienert & Rahmstorf, 2012; Krasting et al., 2016; Landerer et al., 2007; Lorbacher et al., 2010; Pardaens et al., 2011; Yin et al., 2010; Yin & Goddard, 2013).

**Figure 4:**
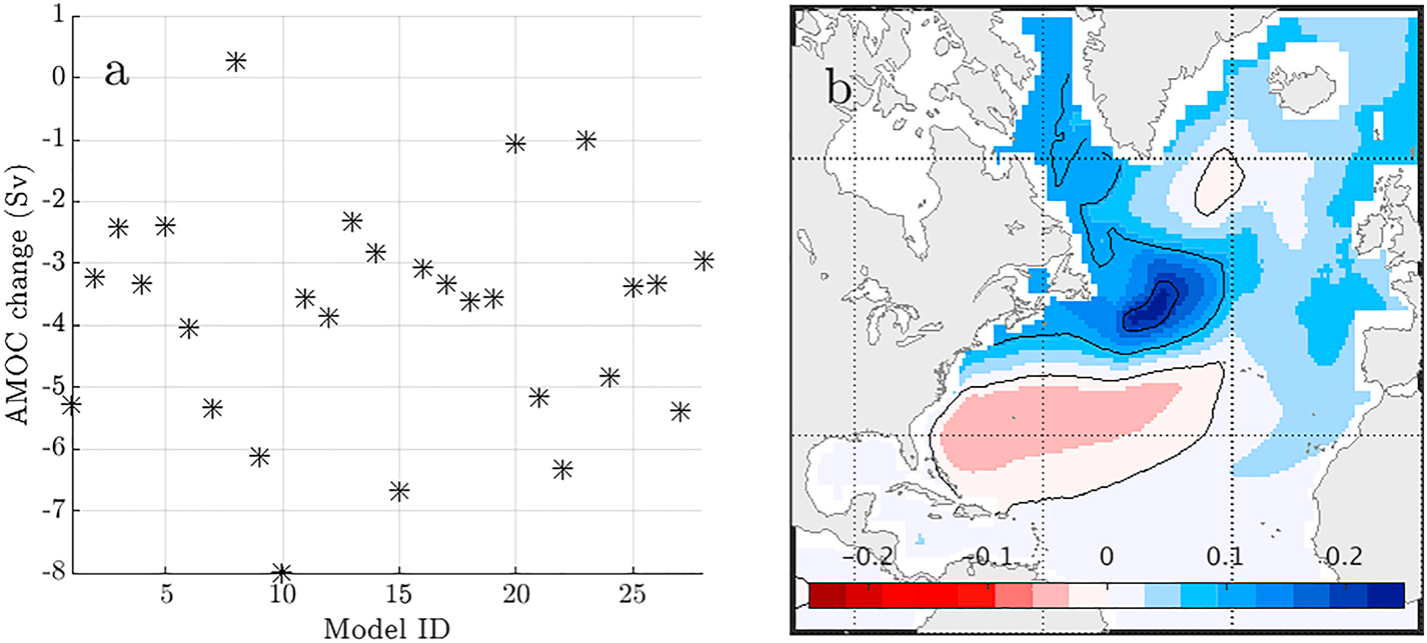
(a) Change in maximum AMOC strength for a 28 Coupled Model Intercomparison Project Phase 5 model, RCP4.5‐forced, ensemble, from 1976–2000 to 2076–2100, as calculated by Chen et al. (2018). (b) Ensemble mean dynamic sea level change (m) from 1976–2000 to 2076–2100.

The AMOC weakens over the 21st century in most CMIP3 and CMIP5 simulations (Church et al., 2013), with a rate that varies widely across emissions scenarios and models (e.g., Figure 4a; Bakker et al., 2016; Cheng et al., 2013; Heuzé, 2017; Huber & Zanna, 2017; Schleussner et al., 2011; Weaver et al., 2012).

The amplitude and spatial pattern of DSL changes associated with 21st century AMOC weakening has been noted in several studies (Schleussner et al., 2011; Yin et al., 2009). However, such studies have generally considered the ensemble mean DSL change (Figure 4b), or a small subset of available models, and have often focused on the Northeast United States only, limiting analysis of intermodel or regional differences.

An assessment of the robustness of the scaling of North Atlantic DSL to AMOC change across climate models is missing in the literature. To fill this gap, we perform a brief analysis using available datasets, including the results of Chen et al. (2018), who investigated the relationship between 21st century changes in DSL and the annual‐mean maximum AMOC stream function below 500 m in a large (30‐member) CMIP5 ensemble. Models included in this ensemble show an AMOC decline from 1976–2000 to 2076–2100 ranging from approximately zero to 8 Sv (Figure 4a).

**Figure 5:**
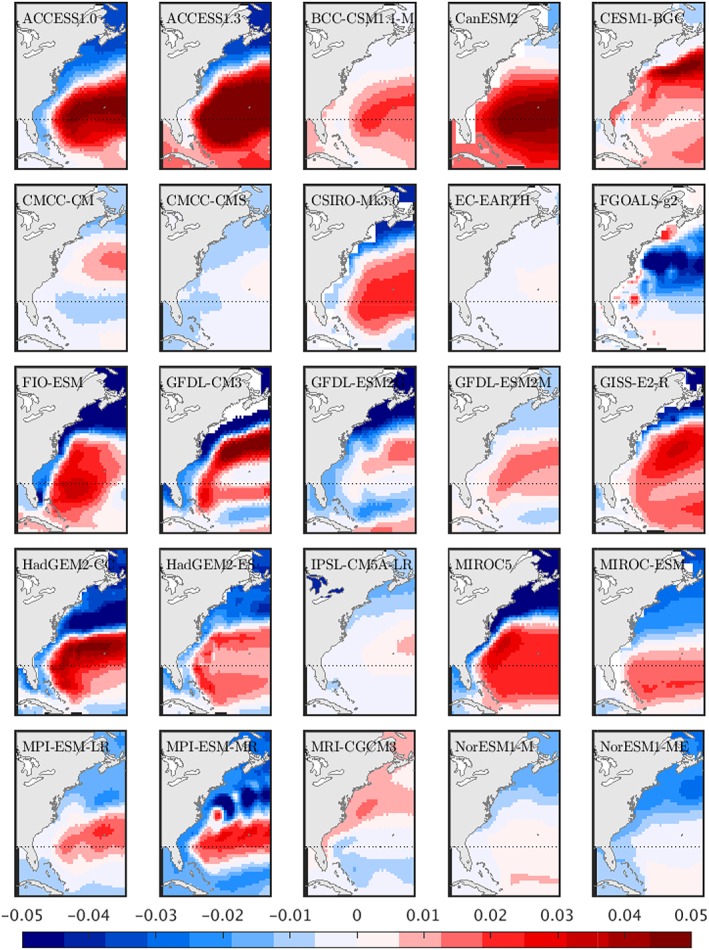
Map of the ratio of dynamic sea level change to AMOC change (m/Sv; 2076–2100 minus 1976–2000) for 25 RCP4.5‐forced Coupled Model Intercomparison Project Phase 5 models with AMOC weakening larger than 2 Sv.

In Figure 5, we calculate the AMOC‐DSL scaling coefficient for 25 CMIP5 models over this century‐long period, at a 1° horizontal resolution.

There are broad similarities in the spatial pattern of scaling coefficients and that of the ensemble mean DSL change (Figure 4b), with only a few models showing dramatic differences from the subtropical high/subpolar and coastal low relationship (e.g., MRI‐CGCM and FGOALS‐g2). However, the amplitude of the scaling coefficient near the U.S. East Coast ranges widely, both north and south of Cape Hatteras, across the ensemble, along with substantial meridional gradients along these coastal regions within individual models.

The diversity of model‐specific scaling coefficients along the western boundary can also be shown with a regression of DSL change against AMOC change, that is,

(7) $$\text{ΔDSL}\left(x,y,m\right)=\alpha \left(x,y\right)\text{ΔAMOC}\;\left(m\right)+\;\varepsilon \left(x,y,m\right)$$

where *x* and *y* are longitude and latitude, *m* is the model index, *α* is a local scaling coefficient, and ε is a residual. (Although the RCP 4.5 scenario is shown, spatial patterns of DSL change, and DSL change associated with AMOC change, do not exhibit strong RCP‐dependence; Chen et al., 2018; Little, Horton, Kopp, Oppenheimer, & Yip, 2015; Yin, 2012; Yin et al., 2009).

**Figure 6:**
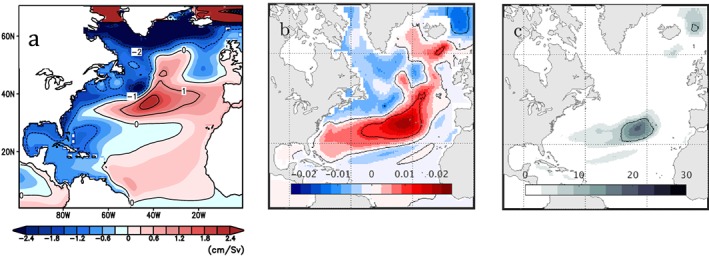
(a) From Woodworth et al. (2014). Regression coefficients of annual mean sea level and overturning transport (at the same latitude) for depths between 100 and 1,300 m using a 1° ocean model, for the period 1950–2009, without wind forcing. (b) Linear regression coefficient (*α*) of DSL change against the change in maximum AMOC strength for the models shown in Figure 5 (m/Sv). (c) Variance in DSL change explained by AMOC change (%). DSL = dynamic sea level; AMOC = Atlantic Meridional Overturning Circulation.

Local regression coefficients, shown in Figure 6b, indicate a meridional tripole in the North Atlantic; models with more AMOC weakening are associated with larger DSL rise in the subtropical gyre and larger DSL fall in most of the subpolar gyre and the tropics. This pattern bears some similarity to the dominant mode of sea surface height variability over the historical record (e.g., Hakkinen & Rhines, 2004; Yin & Goddard, 2013), the multimodel mean 21st century change observed in CMIP simulations (Figure 4b; Little, Horton, Kopp, Oppenheimer, & Yip, 2015; Yin, 2012; Yin et al., 2009, 2010), and a regression of DSL on AMOC strength in a model simulation of the historical period (Figure 6a). Coastal regression coefficients range from approximately −1.5 to 0 cm/Sv, with more negative values in U.S. East Coast regions north of Cape Hatteras.

However, regression coefficients in Figure 6b diverge from those obtained through a regression of annual mean DSL on AMOC over the 1950–2009 period (Figure 6a) and those predicted by equation (6), particularly along the western boundary, where the CMIP5‐derived pattern does not show a universal anticorrelation of sea level and the AMOC. (We note that the single‐valued AMOC index used in Figure 6b is different than the meridionally varying index used in Figure 6a. However, we would not expect this to affect the *sign* of regression coefficients, if AMOC transport changes are meridionally coherent). Perhaps more important than the spatial pattern in Figure 6b is the fact that only a small fraction of intermodel DSL variance is explained by differences in AMOC strength change (Figure 6c). In coastal regions, and in the subpolar gyre, factors unrelated to AMOC strength are principally responsible for the wide spread in 21st century projections of U.S. East Coast DSL rise (Yin et al., 2009, 2010; Kopp et al., 2014; Little, Horton, Kopp, Oppenheimer, & Yip, 2015; Minobe, 2017).

It is possible that differences between Figures 6a and 6b originate in a timescale‐dependent relationship. This was suggested by Yin and Goddard (2013, their Figure 3) based on (1) similarities between the DSL patterns of observed decadal trends and 21st century model trends; (2) similarities between observed and modeled Empirical Orthogonal Function (EOF) patterns (describing interannual variability); and (3) differences between DSL patterns associated with long‐term trends and interannual variability. Similar conclusions were drawn by Lorbacher et al. (2010). Model‐derived scaling coefficients for interannual AMOC‐DSL relationships north of Cape Hatteras appear to be more consistent than those in Figure 5, and more consistent with the theoretical values in section 2. For example, Bingham and Hughes (2009) find a scaling of ‐1.7 cm Sv^−1^, Woodworth et al. (2014) find ‐1.5 cm Sv^−1^, and Little et al. (2017) obtain −1.8 cm/Sv. However, the wide spread in scaling coefficients across models under identical forcing (Figure 5) suggests that differences in model representation are critical over longer timescales. Although an analysis of these regional and inter‐model differences is beyond the scope of this review, we highlight its importance and discuss possible explanations in sections 6 and 7.

## 5 Evidence of an AMOC‐DSL Relationship in Observations

The first direct, continuous, basin‐wide, observations of the AMOC began in 2004 with the RAPID project (Rapid Climate Change; Cunningham et al., 2007). This record is now complemented by two other basin‐wide in situ programs: NOAC at 47°N (North Atlantic Changes; Mertens et al., 2014) and OSNAP, around 60°N (Overturning in the Sub‐polar North Atlantic; Lozier et al., 2017). Although RAPID observations have revealed a wealth of information, they provide only a 13‐year time series at 26°N at time of writing. This limited record hinders an observation‐based assessment of AMOC‐DSL relationships, especially over the decadal and longer timescales of primary interest here. Over shorter timescales, Ezer (2015) compared monthly RAPID observations to the Atlantic City‐Bermuda tide gauge sea level difference, finding a correlation of 0.27. In the same analysis, Ezer noted substantial differences in correlations, and lag/lead relationships, between the sea level difference and the three individual components of AMOC observed by RAPID (Ekman, Florida Current, and Mid‐Ocean transport). Piecuch et al. (2019) also note differing relationships between each of these AMOC components and New England coastal sea level, with only the Ekman component exhibiting strong coherence.

In addition to the RAPID record, longer observations of elements of the North Atlantic circulation are available: for example, the Florida Current time series since 1982 (Meinen et al., 2010), the Oleander time series of Gulf Stream transport since 1992 (Rossby et al., 2014), and the position of the Gulf Stream Extension since 1955 (Joyce & Zhang, 2010). Studies based on models (e.g. Saba et al., 2016; Sanchez‐Franks & Zhang, 2015) and observations (e.g., Kopp, 2013; McCarthy et al., 2015; Park & Sweet, 2015) have shown strong statistical relationships between US east coast sea level and these metrics at up to multidecadal timescales. We consider evidence in this section for relationships between DSL and these and other elements of the North Atlantic circulation, while emphasizing that changes in the latter do not necessarily imply changes in AMOC, as defined in section 2. We briefly discuss the nature of potential linkages with AMOC in section 6.3.

### 5.1 Linkages Between DSL and AMOC Components

The relationship between coastal DSL and the Gulf Stream has been assessed using theory, observations, and models. Studies have considered the roles of Gulf Stream transport, velocity, and position, both upstream and downstream of the detachment at Cape Hatteras, as well as the strength of the Florida Current.

Early studies focused on the relationship between tide gauge observations and the Gulf Stream over seasonal timescales between Florida and Cape Hatteras. Two linkages between ocean circulation and DSL were considered: the cross‐stream (shelf) sea level gradient, related to ocean circulation via geostrophy, and the downstream (along‐coast) sea level gradient, related via the Bernoulli principle. Montgomery (1941) found little evidence for a relationship of the downstream sea level gradient to velocity (in the Gulf Stream).

Attempts to relate the cross‐stream gradient to Gulf Stream fluctuations were more successful. By examining tide gauges along the Florida coastline, and between Charleston and Bermuda, Montgomery (1938) concluded that fluctuations in Gulf Stream strength could be seen in cross‐stream sea level measurements. The study of Iselin (1940) supported the utility of tide gauges south of Cape Hatteras (Key West to Charleston) for estimating the Gulf Stream strength. Both studies were based on comparison with shipboard hydrography and related a summer‐to‐fall increase in sea level to a drop in Gulf Stream transport. Hela (1951) revisited the two earlier studies to relate the annual cycle of sea level difference from Miami to Cat Cay, Bahamas to transport estimates of the Gulf Stream from ship drift (Fuglister, 1948), finding a high correlation (*r* = 0.95) between the zonal sea level gradient and meridional transport in the Gulf Stream. Blaha (1984) removed local effects of the inverse barometer, seasonal steric effects, river runoff, and local wind stress, to demonstrate that the residual sea level variability had a robust correlation with Gulf Stream transport on seasonal timescales. More recently, Park and Sweet (2015) found an interannual‐ to decadal‐timescale relationship between Florida Current transport and tide gauge observations at three locations in Florida using empirical mode decomposition, with a scaling coefficient determined to be consistent with geostrophic balance.

Similar techniques have been used to examine links between Gulf Stream transport variability and sea level in the Mid‐Atlantic Bight. Ezer (2013) found a longer‐period relationship between Mid‐Atlantic Bight DSL and the sea surface gradient across the detached Gulf Stream. The offshore DSL gradient was found to be correlated with sea level at individual tide gauge locations over decadal timescales, as suggested by Yin and Goddard (2013). However, the robustness of these longer period relationships, found using statistical techniques including empirical mode decomposition, has been questioned (Chambers, 2015). Model‐based support for observed Florida Current and Gulf Stream correlations is stronger on short timescales: for example, while idealized modeling studies show that an oscillatory transport of Gulf Stream is associated with coherent coastal sea level variations along the southeast U.S. coast (Ezer, 2016), Woodworth et al. (2017) do not see evidence of Florida Current transport variations in annual mean sea level, either averaged south of Cape Hatteras or in the difference of sea level averaged over the coastline north and south of Cape Hatteras.

Coastal sea level has also been related to the position of the Gulf Stream on leaving the coast at Cape Hatteras, known as the Gulf Stream North Wall (GSNW; Fuglister, 1955). Indices of the GSNW based on sea surface temperature exist since 1966 (Taylor & Stephens, 1980) and based on temperature at 200 m since 1955 (Joyce & Zhang, 2010). The GSNW has been shown to exhibit quasi‐decadal fluctuations that are similar to those in sea level data along the U.S. East Coast (McCarthy et al., 2019; Nigam et al., 2018). Kopp (2013) found a significant antiphase relationship between the GSNW index and DSL north of Cape Hatteras and a likely in‐phase relationship between GSNW and DSL south of Cape Hatteras. McCarthy et al. (2015) noted the difference of sea level south and north of Cape Hatteras projected onto the surface velocity of the GSNW. Whether these sea level variations reflect AMOC strength changes relies upon an understanding of the interaction of different AMOC components: Early explanations associated an AMOC strengthening with a northward shift in the GSNW (e.g., Eden & Jung, 2001). However, recent literature indicates the inverse; AMOC strengthening drives a southward shift in the GSNW due to coupling between the Gulf Stream, Deep Western Boundary Current, and topography (Joyce & Zhang, 2010; Sanchez‐Franks & Zhang, 2015; Yeager, 2015; Zhang & Vallis, 2007).

AMOC variability may also be related to heat content and density variations in the subtropical and subpolar gyres (Williams et al., 2014). Such changes in gyre properties have been found to be correlated with U.S. East Coast sea level changes (Thompson & Mitchum, 2014). Frederikse et al. (2017) find that, after being adjusted for local atmospheric (wind and pressure) effects and smoothed on decadal timescales, sea level changes from tide gauges north of Cape Hatteras over 1965–2014 are correlated with upper‐ocean steric height changes in the Labrador Sea and the deep midlatitude North Atlantic intergyre region. This is consistent with the strong relationship between U.S. coastal sea level and Labrador Sea level in the CMIP5 ensemble (Minobe et al., 2017).

Other studies have considered property differences between gyres, in particular the meridional density gradient, as an indicator of AMOC strength (Butler et al., 2015; De Boer et al., 2010; Kienert & Rahmstorf, 2012; Rahmstorf, 1996; Rahmstorf et al., 2015; Sijp et al., 2012; Thorpe et al., 2001). The meridional density gradient can be related to the gyre‐scale sea level gradient, which has been shown to be related to the strength of the AMOC over sufficiently long timescales (multidecadal and longer; Butler et al., 2015). This relationship was investigated by McCarthy et al. (2015), who used differences in DSL north and south of Cape Hatteras as an estimate of the meridional density gradient between the subtropical and subpolar gyres. The meridional gradient projected strongly onto the circulation in the intergyre region and changes in the subpolar heat content on interannual to decadal timescales. Output from a NEMO 0.25° simulation related the differences in DSL north and south of the modeled Gulf Stream separation to the meridional heat transport at 40°N, indicating a relationship to AMOC.

## 6 Possible Sources of Regional, Intermodel, and Model‐Observational Discrepancies

The diagnostic geostrophic relationship between AMOC transport and U.S. East Coast sea level derived in section 3 implies a scaling coefficient of order −1 to −2 cm/Sv with little alongshore variation. Although some numerical simulations find coefficients within this range over portions of the U.S. East Coast, a uniform along‐coast scaling of AMOC strength and DSL is not evident (section 4). These deviations from theory likely result from neglect of terms in the more complete zonal momentum balance (e.g., friction, nonlinearities, time dependence), or a breakdown in the assumption that U.S. East Coast sea level is related to the depth‐averaged boundary pressure via a constant effective layer thickness (***H***_***e***_ in equation (6)). Similarly, intermodel differences under identical forcing must originate in the relative magnitude of neglected dynamical terms, and their treatment in models. Observations of other components of the North Atlantic circulation offer general support for antiphase relationships between large‐scale meridional transport and DSL along portions of the U.S. east coast but are constrained by their limited record length and indirect relationship with AMOC (section 5).

In this section, we highlight findings from three areas of research that can at least partially account for these regional, intermodel, and model‐observational discrepancies via: (1) friction and bathymetry at the coast, (2) local forcing, and (3) temporal and spatial incoherence of AMOC and its components. In section 7, we suggest opportunities to better integrate these findings into the sea level literature.

### 6.1 Friction and Topographic Influence on Coastal Sea Level

Most analyses noted in sections 4 and 5 interpret AMOC‐DSL relationships based on geostrophy. To understand offshore influences on coastal sea level, however, requires addressing ageostrophic flows and forcing on the slope and shelf, where water column thickness goes to zero and friction is important.

Recently, Minobe et al. (2017) have addressed coastal DSL onshore of a western boundary current using a reduced gravity, vertical sidewall model. Such a framework bears similarity to that used in other studies of remotely forced coastal sea level variability in western boundary regions (e.g., Hong et al., 2000; Thompson & Mitchum, 2014). In this model, interior DSL gradients are moderated by friction within a coastal boundary layer. Their main result can be written as

(8) $$\frac{h_{W}}{f}={\left(\frac{h_{W}}{f}\right)}_{0}+\int_{y}^{y_{0}}\frac{\beta h_{I}}{f^{2}}\;dy^{\prime }$$

where *h*_*W*_ and *h*_*I*_ are sea level as a function of latitude (*y*) at the western boundary and in the ocean interior respectively (*h*_*I*_ is taken near the boundary, but to the east of any western boundary current.) The integral is from the *y* value of interest to a reference point y_0_ further north, where $\frac{h_{W}}{f}={\left(\frac{h_{W}}{f}\right)}_{0}$. The western boundary sea level is determined from a combination of the interior ocean sea level and the sea level from higher latitudes; coastal sea level anomalies are smaller than those in the interior and shifted toward the equator for reasons which become clearer if the vertical sidewall case is seen as a limiting case of a sloping continental shelf and slope.

The southward shift and weakening of the “interior” sea level signal as it approaches the coast is reminiscent of the linear, barotropic case with a sloping sidewall as explored by Becker and Salmon (1997) following the ideas of Welander (1968). Instead of being controlled by contours of constant *f*, as in the flat‐bottom case, the flow is controlled by contours of constant *f/H*, where *H* is the ocean depth. With varying bathymetry, the subpolar gyre intrudes between the coast and the extension of the subtropical gyre, resulting in a reversing pattern of currents along the continental slope, rather than a simple single‐signed western boundary current. Similar behavior is found in highly nonlinear and baroclinic cases with a sloping sidewall (e.g., Jackson et al., 2006, their Figure 1). Sea level signals from the interior therefore appear further equatorward at the coast.

**Figure 7:**
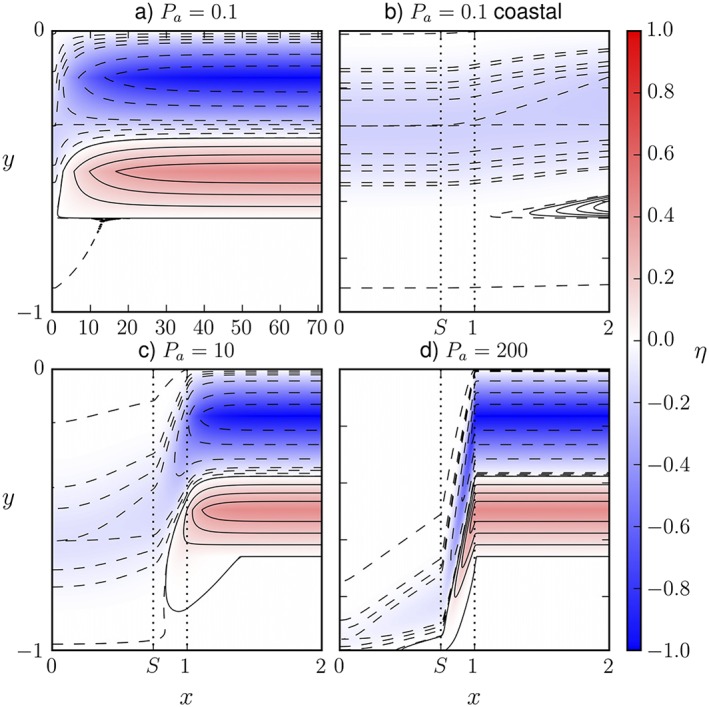
From Wise et al. (2018). Sea level contours (nondimensional; dashed negative) for a given idealized coastal bathymetry along the western boundary of an ocean basin, where *x* and *y* are the nondimensional across‐shore and alongshore coordinates, respectively. Vertical dotted lines indicate the continental shelf break at *x* = *S* and continental slope floor at *x* = 1. Panels show sea level patterns for different Péclet numbers: (a) *P*_a_ = 0.1, (b) *P*_a_ =0.1, (c) *P*_a_ =10, and (d) *P*_a_=200. Panels (b)–(d) show only the coastal region.

Wise et al. (2018) assess the influence of continental shelf bathymetry, using linear dynamics and ocean bottom pressure as the central variable (equivalent to sea level in a single layer case). In this formalism, sea level is “advected” along contours of *gH/f* (such contours can be thought of as representing the stream function of a fictitious flow carrying the sea level signal toward the coast at a speed which becomes the long Rossby wave speed over a flat bottom). The “advection” is toward the west and then toward the equator along the slope, in competition with a “diffusion” by bottom friction (Figure 7).

As the coast is approached, the geostrophic shoreward flow becomes balanced by an offshore flow in the bottom Ekman layer, as in Csanady (1978). Friction is required for alongshore sea level gradients to exist without a flow through the coast, as a purely geostrophic balance would imply.

When shelf bathymetry is included, coastal sea level is still determined by the combination of a poleward reference value, and a weighted integral of interior sea level between that poleward latitude and the latitude of interest. However, the coastal sea level anomaly can be smaller than that predicted in the Minobe et al. (2017) configuration: the western pressure signal can all be on the continental slope, with shallower currents causing it to be cancelled out at the coast. Wise et al. (2018) find that coastal DSL depends crucially on the strength of the bottom friction and the shelf bathymetry. The major dependence is on a nondimensional number, the analogue Péclet number *P*_*a*_ = β*HL*/*r*, where *H* is the offshore layer thickness, *L* is the width of the topography, and *r* is a linear bottom friction coefficient (Figure 7). As friction weakens, the coastal signal shifts further south and becomes weaker compared to the interior sea level.

Equation (8) is a limiting case for a vertical sidewall, in which the solution becomes independent of the strength or form of the friction. In this linear case, the vertical sidewall limit is found to produce the largest coastal signal, for a given upper layer thickness. The mechanism here can be considered to be a breakdown of the assumption that there exists a meaningful effective layer thickness *H*_*e*_. Counterpropagating currents over topography mean the boundary pressure *p*_*W*_ can change sign over the upper continental slope, so equation (5) shows that *H*_*e*_ can become larger than *H*. Thus, the coastal sea level can be smaller than that implied by the depth‐averaged pressure divided by a meaningful effective layer depth. This reduction of the coastal signal can be interpreted as the result of the influence of coastal trapped waves, which carry the interior signal equatorward along the western boundary, as seen for periods of a few days in the model simulations of Ezer (2016). See Hughes et al. (2019) for more detail on the smoothing and “advective” effect of coastal trapped waves on boundary sea level.

We should note, though, that although friction plays a crucial role in communicating sea level changes to the coast, it does so in a manner which does not affect the zonal momentum balance (equation (1)), which remains geostrophic.

### 6.2 Locally (Shelf‐) Forced Sea Level Variability

The presence of locally forced sea level variability along the shelf may interfere with the simple AMOC‐DSL scaling. Similar to section 6.1, ageostrophic dynamics are relevant, although in this case they may also upset the zonal momentum balance.

Local meteorological and terrestrial forcing mechanisms, namely, winds, barometric pressure, and river runoff, have long been shown to drive U.S. East Coast sea level variability. Part of this variability can be static in nature, as with the case of inverted barometer effects related to atmospheric pressure, which are found to contribute sizably to variability at many tide gauges (Piecuch & Ponte, 2015; Ponte, 2006). By definition, static signals are not directly related to circulation changes. As such, their separate treatment, and removal if possible, is useful when assessing the relation between tide gauge and AMOC variability.

Effects of local winds have been extensively examined in the observational studies of Blaha (1984), Andres et al. (2013), Domingues et al. (2018), and others. Simple regression analyses suggest an important contribution of local winds, particularly the alongshore component, to observed tide gauge variability at interannual to decadal timescales. Setup from onshore winds can also contribute to static variability at the coast (e.g., Thompson, 1986), but separate estimation of these effects has not been examined in detail. Recent studies (Domingues et al., 2018; Li et al., 2014; Little et al., 2017; Piecuch et al., 2016; Woodworth et al., 2014) reinforce the importance of near‐coastal winds and barotropic dynamics to explain US east coast tide gauge records over interannual to decadal timescales.

Much less studied has been the effect of river runoff. Meade and Emery (1971) found that about 20–29% of variations in detrended annual mean sea level in U.S. East Coast tide gauges could be accounted for by changes in riverine input. Their results are consistent with the analysis by Piecuch, Bittermann, et al. (2018), who relate sea level signals to the buoyancy‐driven geostrophic coastal currents associated with the runoff. Other studies focusing on different river systems and utilizing different data sets have concluded that riverine input is negligible. For example, Hong et al. (2000) found contributions from runoff to be unimportant relative to winds for tide gauges south of 38°N (see also Blaha, 1984). Calafat et al. (2018) did not find a relationship between river runoff and decadal modulations in the amplitude of the sea level annual cycle along the South Atlantic Bight. However, beyond the few studies noted here, most US east coast sea level studies have ignored riverine effects.

**Figure 8:**
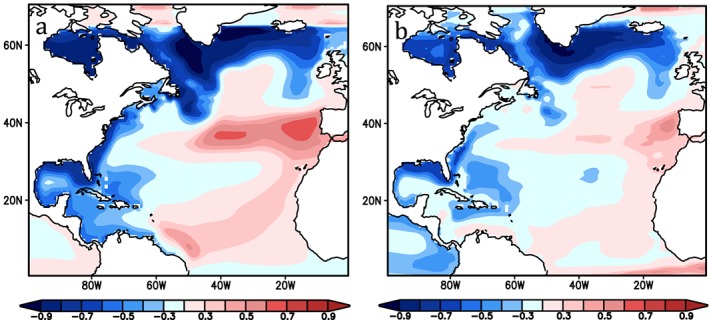
From Woodworth et al. (2014). (a) Correlations of detrended values of annual mean sea level and overturning transport at the same latitude for depths between 100 and 1,300 m using the simulations shown in Figure 6a (without wind forcing). (b) As in Figure 8a, with winds.

Regardless of its origin, the presence of local forcing can lead to large sea level variations that mask the open ocean influence, and thus the emergence of AMOC‐associated sea level variability relative to locally forced variability. For example, correlations of DSL and AMOC are weaker in simulations that include wind forcing, particularly close to the coast and along the Northeast U.S. shelf (Figure 8).

The fact that atmospheric variability has an almost white spectrum means that locally forced variability will tend to be the dominant influence at higher frequencies, with emergence of the open ocean influence at lower frequencies. Little et al. (2017) conclude, using a climate model ensemble, that coherence with AMOC emerges along the northeast U.S. coast at periods of around 20 years. This conclusion echoes Woodworth et al. (2014), who find that local winds dominate nearshore sea level variability on interannual timescales.

We note, however, that local forcing may evolve over longer timescales and may be responsible for some of the model spread seen in Figure 5. For example, Woodworth et al. (2017) suggest that changes in the 20^th^ century wind field may underlie long‐period changes in coastal sea level. Furthermore, atmospheric forcing is spatially coherent over very large scales; changes in local forcing may be associated with large‐scale patterns of change that also influence AMOC and/or remote regions of the ocean.

### 6.3 Spatiotemporal Complexity of AMOC, Hydrography, and Current Changes

The fact that AMOC is the residual of a spatially and temporally complex system of surface and deep currents (Figure 2; see other reviews in this volume) underscores the relevance of the previous two sections for interpretations of observations: any current used as a proxy for AMOC (e.g., the Florida Current) may be characterized by an ageostrophic momentum balance (e.g., due to inertial terms in western boundary currents, or frictional effects in coastal currents). In fact, it is likely that ageostrophic terms become more important at these smaller scales.

An additional important consideration is that currents may be zonally or meridionally compensated, either over shorter timescales, or in the steady state. Observations and modeling studies reveal that changes in AMOC can arise from changes in any of its components, including the interior subtropical gyre (Duchez et al., 2014; Smeed et al., 2018; Zhao & Johns, 2014) and subpolar gyre (Kwon & Frankignoul, 2014; Yeager, 2015), western boundary currents (Beadling et al., 2018; Thomas et al., 2012), and the formation of deep water at high latitude (Medhaug et al., 2011). Additional changes and variability also arise through near‐surface Ekman transports (Kanzow et al., 2007), their barotropic compensation (Jayne & Marotzke, 2001), and eddy transports (e.g., Thomas & Zhai, 2013). All of these exhibit varying degrees of zonal and meridional coherence, reflecting a multitude of forcings occurring over different timescales (Wunsch & Heimbach, 2013).

For example, the Gulf Stream, by which we refer to the full western boundary current near southern Florida, has two branches: the Florida Current and the Antilles Current, which flows offshore of the Bahama Banks (Figure 2). While the Florida Current carries a larger mean transport (about 32 Sv compared with about 5 Sv in the Antilles Current), both exhibit comparable variability (Lee et al., 1996). Thus, the total western boundary current flow could be constant, but its effect on coastal DSL would vary depending upon the respective contributions of the Florida and Antilles currents. In addition, assessing trends in the volume transport of complex, evolving, western boundary currents is challenging. This difficulty underlies the debate surrounding Ezer et al.'s (2013) conclusion that a Gulf Stream decline was responsible for accelerated sea level rise in the mid‐Atlantic Bight (Ezer, 2015; Rossby et al., 2014). The deviation in Gulf Stream transport calculations found across studies is perhaps not surprising, given longitudinally varying changes in the Gulf Stream velocity, width, and position (Dong et al., 2019), and the presence of Gulf Stream meanders, eddies, and recirculation gyres.

Understanding the timescales over which the AMOC indicators discussed in section 5 (e.g., the Gulf Stream, and gyre densities) and AMOC strength variations are coherent is critical to their use as proxies of AMOC. There is little evidence for seasonal and interannual variability of the Florida Current or the Gulf Stream (characteristic over the timescales of many studies cited in section 5) to be related to AMOC. Using evidence that Sverdrup balance holds on multiannual to decadal timescales in the interior subtropics (Gray & Riser, 2014; Thomas et al., 2014; Wunsch, 2011), it can be demonstrated that (subtropical) AMOC variability must be mirrored by changes in the western boundary current at these timescales (de Boer & Johnson, 2007; Thomas et al., 2012). The Gulf Stream can therefore be expected to concentrate decadal‐period changes in both the wind‐driven and the thermohaline circulations, both of which are predicted to weaken in the 21st century (Beadling et al., 2018; Lique & Thomas, 2018; Thomas et al., 2012). However, this finding only applies southward of approximately 35°N, since the ocean to the north is not in Sverdrup balance (Gray & Riser, 2014; Thomas et al., 2014). Furthermore, there is no satisfactory way of defining the boundary between a western boundary current and the ocean interior when the ocean is dominated by mesoscale eddies (Wunsch, 2008). Models and observations also reveal a strong gyre dependence of AMOC changes, with interannual variability dominating in the subtropical gyre and decadal variability in the subpolar gyre (e.g., Bingham et al., 2007; Wunsch, 2011; Wunsch & Heimbach, 2013; Zhang, 2010). Lozier et al. (2010) used a data‐assimilating numerical model to further demonstrate that gyre‐dependent AMOC changes might be important on up to multidecadal periods.

Relatedly, there is evidence that property changes in the subpolar and subtropical gyres may not reflect changes in AMOC over certain timescales. Processes governing ocean density changes in this region on decadal timescales remain unclear (Williams et al., 2015; Buckley & Marshall, 2016; Menary et al., 2015; Piecuch, Ponte, et al., 2017; Robson et al., 2016); remote Rossby wave signals, local atmospheric forcing, changes in deep convection and water mass formation, mean flow advection, and gyre circulation “wobbles” all potentially play a role (Buckley & Marshall, 2016). Although data collected in the subpolar and subtropical gyres suggest southward propagation of deep hydrographic properties on advective (multiannual to decadal) timescales in the Labrador Current and Deep Western Boundary Current of the subtropical gyre (e.g., Molinari et al., 1998; Talley & McCartney, 1982; van Sebille et al., 2011), tracer studies have identified that the majority of water in the Labrador Current does not pass southwards into the subtropical gyre but instead cyclonically recirculates back around within the subpolar gyre (e.g., Bower et al., 2009; Rhein et al., 2002; Zou & Lozier, 2016). Of the deep subpolar water that is advected into the subtropical gyre, the intergyre pathway is not principally via the Deep Western Boundary Current but rather through the interior ocean (Bower et al., 2009; Lozier, 2010; Zhang, 2010), which is compensated by slow upper ocean advective pathways northwards out of the subtropical gyre that reach the greatest transport at depths of approximately 700 m (Burkholder & Lozier, 2011, 2014).

### 6.4 Implications for Along‐Coast Variations and Across‐Model Differences

Collectively, sections 6.1 to 6.3 indicate that U.S. East Coast continental shelf bathymetry, and the evolution of western boundary and coastal currents under local‐ and large‐scale forcing, will influence the local coastal sea level expression associated with a given change in AMOC. The importance of these processes should be expected to vary regionally (e.g., north and south Cape Hatteras, but also within each region); future studies might probe the influence of these smaller scale along‐coast variations on local sea level gradients (see section 7).

Focusing on time‐mean sea level on the shelf, Higginson et al. (2015) suggest that coarse resolution models may exhibit errors in the representation of coastal sea level due to inadequate horizontal resolution, the form of the coastal boundary condition, poor representation of processes in shallow water, and/or unresolved continental shelf atmospheric forcing. Sections 6.1 and 6.2 support the importance of the representation of these coastal processes, and imply that differences in the model resolution may underlie some of the spread shown in Figure 5.

Over the global coastal ocean, Becker et al. (2016) find that climate models have a wide range of success in reproducing the spectral characteristics of observed tide gauge sea level variability. Little et al. (2017) specifically tested the ability of an initial condition ensemble of Community Earth System Model simulations to represent interannual U.S. East Coast DSL variability, finding that Community Earth System Model agrees well with observed tide gauge data along the Northeast U.S. coast, but poorly represents the time‐mean and variability of DSL south of Cape Hatteras. The Minobe et al. (2017) framework (section 6.1) also exhibits disagreement with CMIP5 US east coast DSL changes south of ~35°N (see their Figure 10). This suggests that large‐scale models might be particularly limited in the South Atlantic Bight. Here, in addition to complex shelf bathymetry, DSL variability may also be influenced by incoherence between the Gulf Stream and AMOC, the complex vertical and horizontal structure of western boundary currents, the potential effect of rapid western boundary current flow against the prevailing propagation of information in the direction of boundary waves, and the Antilles Current (section 6.3).

Penduff et al. (2010) find that higher‐resolution models (as fine as 0.25°) show improved representations of variability and time‐mean Sea Surface Height (SSH), especially in the eddy rich regions, in comparison to altimetry. Coastal sea level variability also appears improved with finer resolution, and DSL change under strong external forcing appears to be moderated near the coastline in models of higher resolution (Liu et al., 2016). Other high‐resolution simulations show substantial modification of the coastal sea level signal (e.g., the two MPI models in Figure 5). Such resolution effects deserve more investigation as simulations become available (see, e.g., Haarsma et al., 2016).

In addition to the varied, resolution‐dependent, representation of coastal processes and shelf bathymetry in models, which might be expected to disproportionately affect coastal DSL, the spatial variability in the “interior” DSL change in CMIP5 models implies that more complex changes in the 2‐D overturning, or in the 3‐D structure of the North Atlantic circulation, are relevant for determining patterns of DSL change. Bouttes et al. (2014) suggest that the underlying driver of differences in large‐scale DSL change is related to locations of deep convection. Support for dependence on forcing is also evident in Kienert and Rahmstorf (2012), who find a substantially different DSL response to AMOC changes associated with different forcing (freshwater hosing, CO_2_ increases, Southern Ocean wind stress changes) within the same climate model.

## 7 Perspective and Future Directions

An antiphase relationship between large‐scale North Atlantic meridional volume transport and U.S. East Coast DSL is broadly evident across a range of numerical simulations and observational analyses. This relationship can be interpreted using the simple geostrophic framework introduced in section 3. However, such a framework is insufficient to explain the widely differing along‐coast AMOC‐DSL scalings derived in models and observations, or variation across climate models. Furthermore, such an interpretation limits causal attribution: Geostrophy cannot provide information about the forces that drive sea level changes.

In this review, we have noted some possible origins for regional, model, timescale, and forcing dependence (section 6). However, we are unable to assess the degree to which each is responsible for variations in local scaling coefficients. Explanations for these deviations are essential to improve confidence in reconstructions of North Atlantic variability derived from tide gauge observations or paleoproxies and projections of coastal sea level change from current‐generation climate models.

We thus encourage the sea level research community to pursue the following near‐term goals: (1) an understanding of the relationship between AMOC and other North Atlantic currents; (2) an understanding of the vertical structure of the AMOC and its variation with respect to local bathymetry; (3) an assessment of the importance of ageostrophic processes to AMOC and related currents; and (4) an effort to connect these research results, including their region (latitude‐), model, and timescale dependence, to their origins in heat, momentum, and buoyancy forcing. Such efforts should include new sea level studies, as well as the incorporation of existing and new findings from outside the sea level realm.

A simple step toward the first and second goals involves broadening the features of the ocean circulation analyzed in models beyond a single AMOC metric (e.g., the basin‐wide maximum overturning stream function). Modeled and observed DSL changes have often been compared to AMOC changes at a different latitude, which involves an implicit or explicit assumption that such changes are synchronous and meridionally coherent, which is not supported by the literature cited in section 6.3. Indeed, such a coarse characterization of AMOC may underlie some of the difference in scaling coefficients shown in Figure 5.

As noted in section 4, another critical ambiguity of relevance, particularly important to the interpretation of observational analyses, is the coherence of AMOC and western boundary currents. Other important relationships include those between the GSNW and AMOC; Labrador Sea and subpolar gyre steric changes; and subpolar and subtropical gyre steric changes. Higher‐resolution simulations can now represent the mean state and variability of coastal currents and indicate that climate‐driven changes in these currents may differ from those in the large‐scale (e.g., Saba et al., 2016). Although evidence in section 6.3 suggests that many components of AMOC, and subpolar and Nordic Seas buoyancy variability, may be coherent over multidecadal time frames (Pillar et al., 2016), there is evidence that interannual to decadal variability is not, particularly across the intergyre boundary. Modeling studies examining AMOC‐DSL relationships can easily include metrics of some of these other AMOC components and indicators (possibly over different timescales), which would improve the scope of their results, and the ability to reconcile with observations.

The direct observational record of AMOC variability is limited; in this review, we have focused on the longer observed record of AMOC components. However, the ever extending record of AMOC at 26°N is now complemented by the OSNAP array, providing some perspective on gyre dependence, the meridional coherence of AMOC, and the relationship with other AMOC components. These AMOC records are complemented by new observational campaigns over the U.S. East coast continental shelf and slope (e.g., Andres et al., 2018, Gawarkiewicz et al., 2018). In addition to these instrumental records, proxy records of both coastal sea level and AMOC are available that are able to resolve decadal‐centennial fluctuations (Engelhart & Horton, 2012; Kemp et al., 2017, 2018; Rahmstorf et al., 2015; Thornalley et al., 2018). Complemented by model results, these proxy observations could provide valuable constraints on multidecadal to centennial AMOC‐DSL covariability.

With respect to the assessment of ageostrophic processes, we note that many modeling centers have begun to provide the output required to compute closed momentum budgets offline (Gregory et al., 2016; Wunsch & Heimbach, 2009; Yeager, 2015). Such budgets, both zonally integrated, and local, would clearly indicate the importance of ageostrophic processes, their time and latitude dependence, and (if possible) differences across a set of models. They could also include the effect of terms, including nonlinearity (Hughes et al., 2019), and, in higher‐resolution models, eddy variability (Grégorio et al., 2015; Sérazin et al., 2015), that are not discussed in this review. High‐resolution models also offer promise for better resolving the shelf and shelf processes, and they may constitute a means for testing the theories of coastal modulation of interior signals (section 6.1), under a wider range of conditions, forcing, and timescales.

Such analyses also move beyond the purely diagnostic, degenerate, statement of force balance supplied by geostrophy, allowing an understanding of the local, regional, basin, and global scale forcing responsible for coastal sea level changes. The incomplete interpretation provided by geostrophy is evident in Goddard et al. (2015), who linked an “extreme” interannual sea level rise event in the northeast US with an abrupt 30% AMOC weakening. However, this event occurred coincident with an anomalously negative North Atlantic Oscillation (NAO) associated with atmospheric pressure and wind anomalies. Piecuch and Ponte (2015) and Piecuch et al. (2016) demonstrated that 50% of this event could be explained by the inverse barometer effect and the remainder could be partly explained by local winds. The 30% drop in the AMOC itself was observed in the Gulf Stream transport (Ezer, 2015) and was explained by wind forcing (Zhao & Johns, 2014). It is thus more appropriate to view the sea level anomaly as driven by all of the forcings (local and remote) associated with the extreme NAO anomaly. Over longer timescales, causality often remains unclear: for example, differences in observed sea level changes along the US east coast have been attributed to changes in Gulf Stream position and strength, AMOC strength, and steric changes. While these changes may be coupled, and serve as indicators of AMOC, they do not identify causal drivers.

Even if causality can be established under certain forcing and timescales (e.g., interannual, driven by NAO), it does not imply that the same processes and AMOC components (and sea level signatures) are always relevant (e.g., on centennial timescales in the past or future). For example, Kenigson et al. (2018) find that the relationship between DSL and NAO is nonstationary, echoing the results of Andres et al. (2013). Looking farther into the future, 21st century changes in AMOC strength in climate models are principally forced by greenhouse gas‐associated heat and buoyancy fluxes in the North Atlantic (Beadling et al., 2018; Bouttes et al., 2014; Slangen et al., 2015), rather than NAO‐associated wind stress.

Separation of local and remote wind‐driven changes in circulation and sea level from remote buoyancy/deep water driven AMOC changes remains a key challenge. Such work will have to illuminate the timescales and climate forcing under which wind and buoyancy forcing are coupled. For example, Woodworth et al. (2014) indicate that wind forcing alone is largely responsible for decadal timescale sea level variability. However, since this study used a standalone ocean model, it is not clear what processes produce low‐frequency wind variability. Furthermore, the large spatial scales of atmospheric forcing challenge efforts to isolate the AMOC‐forced or remotely forced component of sea level change. Adjoint analyses or perturbation experiments (Heimbach et al., 2011; Pillar et al., 2016; Yeager & Danabasoglu, 2014) may help isolate the roles of wind and buoyancy forcing and elucidate the relevant pathways, state variables, and adjustment processes mediating connections between the open ocean and observed and projected US east coast sea level changes.

To conclude, there are many productive areas of research that can help refine our understanding of the relationship between the large‐scale climate, AMOC, and coastal sea level. Given their importance to future sea level changes on the U.S. East Coast, and reconstruction of preinstrumental ocean circulation and climate variability, we anticipate the research community will pursue them with vigor.
